# Exposure-aware multi-omics and artificial intelligence for biomarker discovery and precision prevention in diffuse glioma

**DOI:** 10.3389/fimmu.2026.1874861

**Published:** 2026-08-21

**Authors:** Suming Zhang, Zhilin Si, Na Yang, Xin Chen

**Affiliations:** 1 Department of Radiology, Key Laboratory of Obstetric & Gynecologic and Pediatric Disease and Birth Defects of Ministry of Education, West China Second University Hospital, Sichuan University, Chengdu, Sichuan, China; 2 Department of Clinical Laboratory, Suining First People’s Hospital, Suining, Sichuan, China

**Keywords:** artificial intelligence, diffuse glioma, exposome, glioblastoma, multi-omics, neuroimmunology, precision prevention, tumor microenvironment

## Abstract

Diffuse gliomas are now diagnosed and studied through integrated molecular classification, radiomics, single-cell biology, spatial profiling, proteogenomics, metabolomics, and artificial intelligence. Yet many precision-medicine models still begin at diagnosis and emphasize tumor-intrinsic molecular features, leaving environmental, occupational, lifestyle, microbiome, metabolic, immune, and treatment-related exposures at the margins. This review develops an exposome-informed view of diffuse glioma biomarker discovery. Biomarker discovery is separated from clinical prevention: current evidence does not justify population-level glioma screening based on environmental exposures, but it does support systematic integration of external exposures and internal exposure-related molecular states with tumor and host biology. The synthesis focuses on five linked dimensions: the limits of current artificial intelligence and multi-omics models when exposure biology is excluded; glioma-relevant exposure domains stratified by evidence strength and measurability; genotoxic, epigenetic, vascular, neuroimmune, and immunometabolic conduits through which exposures may shape tumor ecology; computational strategies for temporally anchored integration of geospatial, occupational, clinical, liquid-biopsy, imaging, tumor-omic, single-cell, spatial, microbiome, and metabolomic data; and clinically realistic applications in high-risk surveillance, recurrence-aware monitoring, treatment-toxicity reduction, and biomarker-guided trial stratification. By aligning exposome science with systems neuro-oncology, the review outlines a translational agenda for exposure-aware glioma biomarkers while maintaining a conservative boundary between established evidence, mechanistic hypotheses, and future clinical implementation.

## 1 Introduction

Diffuse gliomas are uncommon compared with many systemic malignancies, but they impose a large clinical burden because they arise in functionally essential brain tissue, infiltrate beyond radiographic margins, recur frequently, and remain difficult to cure. Contemporary epidemiologic and clinical summaries show that glioblastoma remains the dominant malignant adult diffuse glioma and one of the most lethal primary brain tumors (1, 2). The 2021 World Health Organization classification placed molecular alterations at the center of diagnosis, and current EANO guidance similarly emphasizes integrated histologic and molecular diagnosis, maximal safe resection, radiotherapy, chemotherapy, and individualized clinical decision-making (3, 4). The Stupp regimen established radiotherapy with concomitant and adjuvant temozolomide as a treatment backbone, and MGMT promoter methylation remains the clearest example of a clinically useful predictive biomarker in glioblastoma (5–7).

The molecular era has transformed the field. TCGA defined core pathways in glioblastoma, later genomic studies refined the somatic landscape, and integrated molecular profiling separated diffuse gliomas into biologically coherent progression routes (8–10). IDH, 1p/19q, TERT promoter status, MGMT promoter methylation, histone alterations, and DNA methylation class now anchor diagnosis, prognosis, and treatment interpretation (6–14). Single-cell and spatial studies have further shown that glioma is not a static mass of malignant cells but an evolving ecosystem of plastic malignant states, vascular niches, immune compartments, and bidirectional tumor-host interdependence (15–20).

This biology makes another narrow prognostic-signature synthesis inadequate. The same diagnostic label can contain different malignant cell programs, immune niches, vascular states, metabolic dependencies, and treatment-induced trajectories. Conversely, the same external exposure may not have the same effect across IDH-mutant astrocytoma, oligodendroglioma, pediatric-type high-grade glioma, and IDH-wildtype glioblastoma. Two simplifications should therefore be avoided. Glioma should not be reduced to a purely genetic disease, and environmental risk should not be reduced to a single exposure-outcome association. A more defensible position is systems neuro-oncology, in which exposure data are interpreted through the host and tumor mechanisms that make them biologically plausible.

Despite these advances, precision-medicine models in glioma remain predominantly tumor-centric. They start when tissue is obtained, prioritize tumor-intrinsic molecular features, and often treat external exposures, internal metabolism, immune history, geography, occupation, medication, diet, microbiome, and treatment-related stress as background variables. This is understandable because glioma is rare, latency is long, exposure measurement is difficult, and many environmental associations are modest or inconsistent. The limitation is now visible. Molecular classification explains much, yet it does not fully explain why tumors with similar markers diverge in immune state, metabolic phenotype, recurrence timing, treatment tolerance, or survivorship burden. A broader framework is needed.

The exposome provides a way to widen this view without abandoning molecular precision. Introduced to complement the genome, the exposome captures the totality of environmental exposures across the life course, including external exposures and their internal molecular consequences (21–23). Exposome science has matured into a measurable field that includes blood exposome databases, exposome-wide association studies, longitudinal multi-omics profiling, early-life exposome signatures, and precision environmental health frameworks (24–29). In diffuse glioma, the immediate objective is not population-level prediction of tumor onset. The more defensible objective is to identify temporally valid and biologically interpretable host-tumor states that improve risk stratification, treatment monitoring, recurrence assessment, immunotherapy enrichment, and prevention of avoidable treatment-related harm.

Glioma is not a classic inflammatory disease, yet its clinical behavior is deeply influenced by immune exclusion, myeloid dominance, steroid exposure, hypoxia, vascular dysfunction, and metabolic competition. The exposome is relevant because many exposures are immunologically and metabolically active even when their direct carcinogenic effect is uncertain. Air pollution, pesticides, diet, sleep disruption, microbiome changes, radiation, infection, and medications can all reshape inflammation and metabolism. The exposome is therefore treated here less as a list of causes and more as a set of perturbations that may leave measurable traces in immune, vascular, metabolic, and epigenetic layers. The synthesis draws on clinical neuro-oncology, molecular neuropathology, environmental epidemiology, exposure science, immunology, metabolomics, microbiome research, radiomics, machine learning, liquid biopsy, and spatial biology. The evidence base was organized by translational function rather than by assay alone: epidemiology identifies exposure domains, exposome science links external exposures to internal molecular signatures, tumor omics defines the disease substrate, single-cell and spatial biology localizes mechanisms, liquid biopsy enables longitudinal monitoring, and computational integration connects these layers to prevention-relevant endpoints.

**Figure 1:**
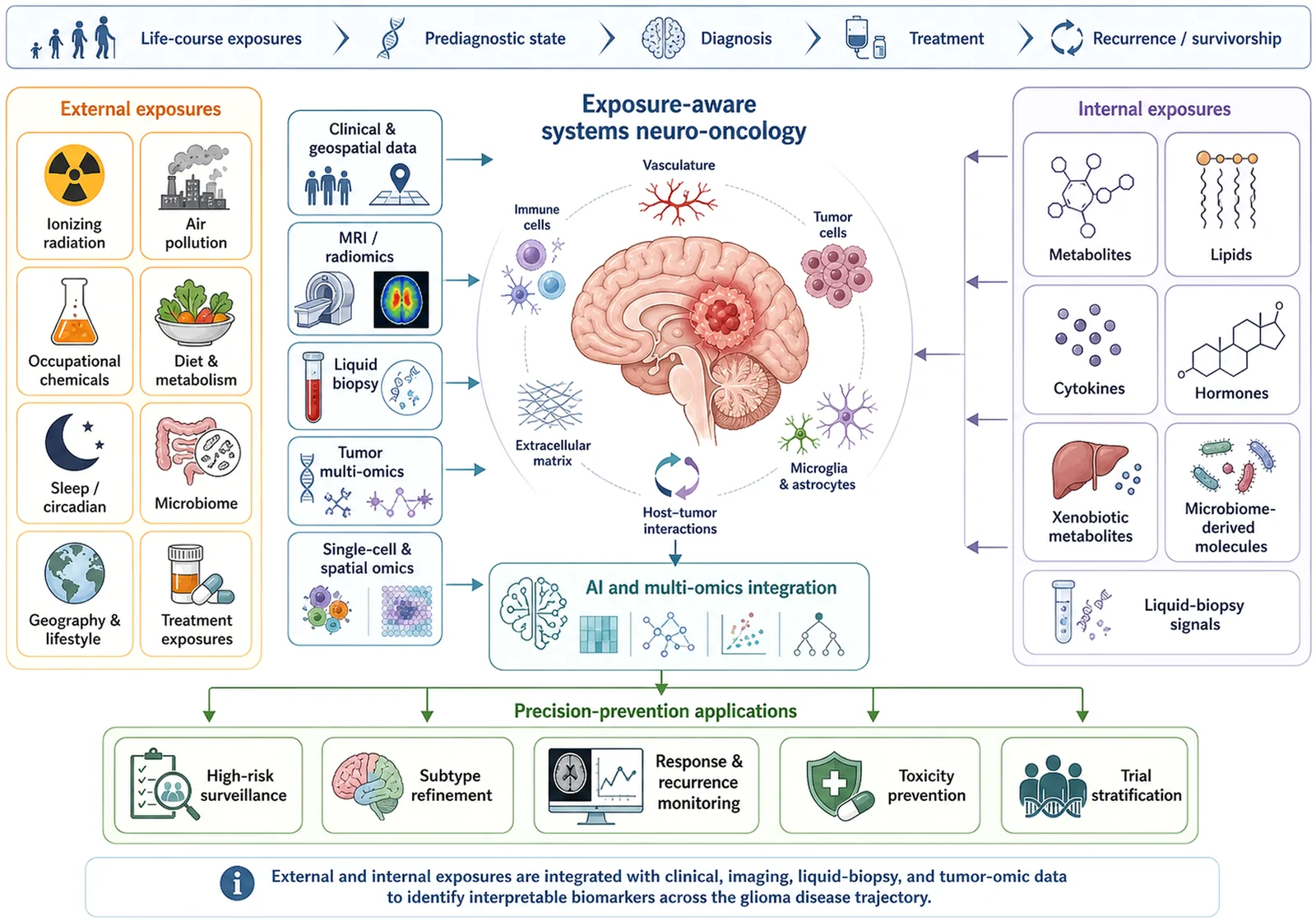
Exposure-aware systems neuro-oncology framework for diffuse glioma. The figure links external exposures, internal exposure biomarkers, host and tumor multi-omics, interpretable artificial intelligence, and prevention-relevant clinical endpoints. The framework emphasizes temporal anchoring across life-course susceptibility, diagnostic state, treatment, recurrence, and survivorship.

Exposure-aware glioma research should move beyond isolated associations toward biologically anchored trajectories. A weak epidemiologic association may still be useful if it points to a measurable internal state, such as vascular inflammation, macrophage-rich hypoxia, lipid remodeling, oxidative stress, steroid-related immune suppression, or treatment-induced metabolic stress. Conversely, a high-dimensional omics signature has limited translational value if it cannot be linked to exposure timing, cell type, tissue niche, liquid biopsy, or a clinical decision. The organizing logic is therefore practical: evidence strength, temporal validity, immune relevance, and clinical actionability matter more than the number of variables assembled in a model. **Figure 1** summarizes the proposed exposure-aware systems neuro-oncology framework.

## 2 Tumor-centered multi-omics models and the missing host-exposure context

### 2.1 Tumor-centered prediction and exposure-aware interpretation

Artificial intelligence and multi-omics have become prominent tools in glioma research. Recent reviews and studies describe artificial intelligence-driven multi-omics approaches, radiomics, radiogenomics, deep learning-based molecular marker prediction, multimodal prognostication, machine learning for neuroimaging, computational neuropathology, and integrated recurrence prediction (30–42). Multi-omics studies have also identified therapeutic networks and master kinases across glioblastoma subtypes, while noninvasive radiomics can infer macrophage infiltration and thus indirectly connect imaging to immune biology (43, 44). These contributions are valuable, but they largely address tumor-centered endpoints such as molecular subtype, survival risk, MRI-based marker prediction, and targetable pathways.

These endpoints are useful, but they are biologically incomplete. A radiomics model that predicts MGMT promoter methylation may be technically impressive, yet MGMT can often be measured directly in tumor tissue. A deep-learning model that predicts survival may not explain whether the adverse trajectory reflects tumor genotype, hypoxia, vascular state, steroid exposure, infection, metabolic stress, or immune suppression. A multi-omics cluster may be biologically interesting but clinically difficult to use if it is not connected to a measurable exposure, modifiable pathway, or surveillance decision. The added value should be a shift from prediction alone to exposure-aware mechanism and prevention.

Exposure-aware precision medicine requires more than a further survival model. It requires exposure domains that can be measured, biological conduits that can be tested, computational integration that respects timing and mechanism, and endpoints that are relevant to surveillance, treatment monitoring, toxicity prevention, and trial stratification.

The limitation is practical as well as conceptual, because the literature is already crowded with survival models that offer limited mechanistic insight. Many such models are difficult to reproduce across institutions because MRI acquisition, segmentation, omics platform, cohort composition, and treatment patterns differ. Exposure-aware models will face the same reproducibility problems, but they also have an additional burden: they must show that exposure variables are measured in a time window that makes biological sense. A geospatial pollution value assigned after diagnosis, a diet questionnaire completed during steroid therapy, or a metabolomic sample collected during infection may be useful for survivorship research but should not be interpreted as an etiologic exposure. Temporal design is therefore as important as algorithm choice, because a biologically misplaced exposure variable can make even a mathematically elegant model misleading.

### 2.2 Immune, metabolic, and liquid-biopsy evidence for host context

The gap is especially clear in glioblastoma immunology. Brain tumors have distinctive microenvironmental landscapes, and glioma cells instruct tissue-invading leukocytes rather than simply coexisting with them (45–47). Single-cell work has identified S100A4 as an immunotherapy target and revealed functional heterogeneity among glioma-associated macrophages (48, 49). Macrophages and microglia in glioblastoma are plastic, therapeutically relevant, and shaped by hypoxia, metabolic rewiring, lipid handling, and cell-cell communication (50–56). These immune states are exactly the kinds of internal biology that exposures could influence, but most current models do not include exposure histories or internal exposure biomarkers.

Metabolism and liquid biopsy expose the same gap. Metabolomic and lipidomic profiling, proteometabolomics of recurrent glioblastoma, longitudinal metabolic remodeling, and glioblastoma metabolomics reviews show that metabolic state changes with grade, recurrence, immune infiltration, and therapy (57–63). CSF and blood-based liquid biopsy can track tumor evolution, extracellular vesicles, circulating biomarkers, and IDH1.R132H-mutant gliomas (64–71). Yet the internal exposome, including metabolites, lipids, microbial products, xenobiotic markers, inflammatory proteins, and treatment-induced biochemical shifts, is rarely treated as a central component of glioma precision medicine.

## 3 The glioma exposome across domains, signatures, and time windows

### 3.1 Defining a measurable glioma exposome

The glioma exposome must be broad enough to capture relevant perturbations but narrow enough to remain analytically useful. Here, the term denotes measurable external, internal, and treatment-related exposure layers that can be temporally linked to glioma susceptibility, tumor-host biology, response assessment, recurrence, toxicity, or survivorship. External exposures include ionizing radiation, air pollution, occupational pesticides and solvents, diet, endocrine and hormone-related factors, radiofrequency fields, plasticizers, combustion products, sleep disruption, geography, and medication or treatment contexts. Internal exposures include metabolites, lipids, proteins, cytokines, hormones, xenobiotic metabolites, microbiome-derived molecules, oxidative stress markers, and liquid-biopsy signals. Treatment-related exposures, including radiotherapy, temozolomide, corticosteroids, antibiotics, antiepileptic drugs, bevacizumab, surgery, infection, and rehabilitation, are treated as a distinct postdiagnostic layer rather than being pooled with prediagnostic environmental risk factors. **Table 1** groups these domains by measurability, biological interface, biomarker layer, translational use, and evidence status so that established radiation effects, heterogeneous environmental associations, and early mechanistic hypotheses are not given the same evidentiary weight.

**Table 1:** Glioma-relevant exposome domains and biomarker interfaces.

Exposure domain	Measurement anchor	Biological interface	Candidate biomarker layers	Near-term translational use	Evidence status
Ionizing radiation	Dose, field, age at exposure, latency, surveillance imaging	DNA damage, repair, chromatin remodeling, vascular injury	Tumor methylation, DNA repair markers, MRI change, plasma proteins	High-risk survivorship surveillance and evaluation of post-radiation lesions	Established environmental risk factor; strongest anchor for exposure-window modeling
Air pollution and traffic mixtures	Residential geocoding, exposure models, traffic proximity, particulate metrics	Inflammation, endothelial dysfunction, oxidative stress, barrier permeability	Inflammatory proteins, perfusion/radiomics, macrophage-rich tissue states	Risk stratification in registries and vascular-inflammatory phenotype discovery	Biologically plausible but epidemiologically heterogeneous; hypothesis-generating for vascular-inflammatory phenotypes
Occupational chemicals and pesticides	Job-exposure matrices, work history, solvent/pesticide exposure, protective practices	Xenobiotic metabolism, oxidative stress, endocrine and immune perturbation	Xenobiotic metabolites, lipidomics, metabolomics, transcriptomic stress modules	Cohort enrichment and biologic dose assessment	Mixed and exposure-specific evidence; best used with internal dose and biologic-response markers
Diet and metabolic state	Dietary pattern, glycemic state, body composition, nutrition during therapy	Systemic metabolism, lipid remodeling, inflammation, microbiome-derived metabolites	Metabolomics, lipidomics, cytokines, stool metagenomics	Supportive-care stratification and treatment-toxicity prevention	Modifiable host state with indirect or inconsistent glioma-risk evidence; stronger for supportive-care and toxicity applications
Immune and endocrine history	Allergy history, hormone exposure, corticosteroids, infection and medication records	Immune tone, myeloid activation, T-cell exclusion, endocrine-metabolic signaling	Cytokines, immune-cell states, steroid-response signatures	Trial stratification and immune-state monitoring	Suggestive immune/endocrine associations; requires cautious interpretation and mechanistic validation
Sleep and circadian disruption	Sleep duration, actigraphy, shift work, treatment-period sleep disturbance	Circadian immune regulation, metabolic stress, glucocorticoid signaling	Inflammatory proteins, metabolomics, activity and sleep features	Postdiagnostic supportive-care intervention and fatigue/toxicity monitoring	Emerging mechanistic and computational evidence; not ready for clinical risk prediction
Microbiome and infection	Stool sampling, antibiotic exposure, infection episodes, microbial metabolites	Microbial immunometabolism, systemic inflammation, treatment tolerance	Metagenomics, metabolomics, cytokines, liquid-biopsy context markers	Immunotherapy correlative studies and toxicity-risk prediction	Emerging immune-metabolic interface; best suited to correlative and longitudinal studies
Treatment exposures	Radiotherapy, temozolomide, steroids, antibiotics, antiepileptics, bevacizumab	Therapy-induced immune suppression, edema, vascular injury, metabolic deterioration	Serial MRI, liquid biopsy, inflammatory proteins, metabolomics	Recurrence interpretation, steroid tapering, infection prevention, survivorship care	Postdiagnostic, directly measurable, and clinically actionable exposure layer

The table prioritizes exposure domains according to measurability, biological plausibility, candidate internal biomarkers, near-term translational use, and evidence status.

This definition also places the internal exposome at the center of the link between exposure science and multi-omics. Residential address, job title, diet, or self-reported sleep provide useful external context but often carry substantial measurement error. Internal molecular layers can capture more proximal biology, including immune activation, vascular injury, lipid metabolism, xenobiotic handling, microbiome-derived metabolites, and treatment-induced stress. They are best handled as candidate biomarkers rather than causal proof, with interpretation depending on timing, confounding, assay reproducibility, and incremental value beyond established molecular classification.

### 3.2 External exposure domains with biological plausibility

Ionizing radiation remains the benchmark environmental exposure in glioma. Adult glioma epidemiology reviews and broader brain tumor epidemiology work consistently recognize radiation as the most established environmental risk factor for glioma and central nervous system tumors (72–75). Radiation-induced gliomas have been reviewed clinically and molecularly, with systematic synthesis suggesting that post-radiation tumors may show distinctive molecular features in some contexts (76, 77). Radiation is therefore an ideal setting for exposome-multi-omics research because exposure timing, dose, field, latency, surveillance imaging, and later tumor tissue may be available. The most realistic application is high-risk survivorship surveillance, not population screening.

Air pollution and traffic-related mixtures occupy a less definitive but biologically plausible position. Studies have examined outdoor air pollution, traffic-related air pollution, and central nervous system tumor risk, including cohort analyses and recent work on ambient outdoor pollution (78–80). The findings remain heterogeneous and potentially confounded, and they do not establish air pollution as a glioma screening variable. Their value in an exposome-multi-omics framework is different: pollution can be geospatially modeled and then related to internal biology such as inflammation, endothelial dysfunction, oxidative stress, perfusion, blood-brain barrier dysfunction, and macrophage recruitment.

Occupational and chemical exposures represent a second heterogeneous domain. Reviews of adult primary brain cancers and environmental risk factors repeatedly note variable but persistent signals around farming, pesticides, solvents, and occupational mixtures (81, 82). Pesticide residue intake from fruit and vegetable consumption has also been evaluated in relation to glioma risk (83). Diet-related evidence is similarly mixed, with systematic dose-response analysis and large prospective cohort work suggesting possible associations while highlighting measurement error and confounding (84, 85). These exposures are most useful when paired with repeated measurement, internal dose markers, and mechanistic validation rather than treated as stand-alone causal explanations.

Modifiable-risk-factor reviews reinforce the same conclusion: the field should be ambitious about measurement but restrained about clinical claims (86). Diet illustrates the problem well. Case-control studies, cohort studies, and Mendelian randomization analyses can each generate signals, including recent work on dietary patterns and glioma risk (87). However, dietary exposure is difficult to measure, changes after symptoms and diagnosis, and is entangled with socioeconomic status, medication, microbiome, and metabolic health. The better strategy is to link dietary patterns to internal metabolites, lipid modules, glycemic or inflammatory markers, and microbiome-derived molecules, then test whether those internal layers map to tumor immune and metabolic states. This turns a weak questionnaire exposure into a biologically testable hypothesis.

Immune, endocrine, and behavioral exposures belong in the same evidence map. Allergy has one of the more consistent inverse associations with glioma risk, although mechanism and clinical actionability remain uncertain (88). That association should not be converted into a claim that immune protection is directly modifiable in patients. Oral contraceptive and hormone-related analyses are informative but do not support simplistic clinical rules (89). Radiofrequency fields and mobile-phone exposure remain important because patients ask about them, but recent systematic reviews, prospective cohort data, and incidence-trend studies do not provide a straightforward causal interpretation (90–92). Childhood brain tumor risk-factor synthesis is relevant for life-course thinking, although pediatric tumor biology differs from adult diffuse glioma (93).

### 3.3 Internal molecular signatures of exposure

Recent exposome-omics studies illustrate how the field can move beyond exposure catalogues, although most of this evidence remains exploratory. A review on the serotoninergic system linked the exposome to brain disorders including glioblastoma, suggesting that neurochemical pathways may provide exposure-sensitive interfaces (94). Integrative multi-omics identified S100A4 as a hub linking DEHP exposure to glioblastoma risk, offering a proof-of-concept for exposure-aware molecular epidemiology (95). Benzo[a]pyrene has been investigated through network toxicology, single-cell transcriptomics, and Mendelian randomization, while multi-omics work has examined sleep deprivation and glioblastoma risk (96, 97). Studies of shared risk factors across brain tumor types further support careful cross-tumor comparison (98). The value of these studies lies in connecting external exposures to molecular networks, single-cell programs, and inferential causal tools; their clinical role in diffuse glioma still requires replication, experimental support, and prospective validation.

### 3.4 Timing windows in susceptibility, treatment, and survivorship

Exposure timing is as important as exposure category. Early-life exposures may shape neurodevelopmental, immune, vascular, or epigenetic states long before a tumor is clinically visible. Adult occupational or residential exposures may be relevant to susceptibility or diagnostic biology if measured before symptoms and treatment. Peridiagnostic exposures, including surgery, steroids, infection, and imaging-related stress, can alter immune and metabolic markers without reflecting etiologic risk. Treatment-period exposures influence response assessment, toxicity, immune competence, and recurrence interpretation. Survivorship exposures affect long-term neurologic, metabolic, infectious, and cognitive outcomes. **Table 2** separates these exposure windows and their interpretation so that etiologic, diagnostic, therapeutic, recurrence-associated, and survivorship-related variables are not collapsed into a single undifferentiated model.

**Table 2:** Exposure windows and interpretive boundaries for exposure-aware glioma research.

Exposure window	Typical data sources	Main biological interpretation	Key confounding risks	Most realistic clinical use
Prediagnostic susceptibility	Radiation history, occupational records, residential geocoding, family history, early biospecimens when available	Etiologic or risk-modifying exposure only if measured before symptoms and diagnosis	Recall bias, residential mobility, socioeconomic status, ancestry, healthcare access, reverse causation	High-risk surveillance research and registry-based risk stratification
Peridiagnostic state	Baseline MRI, surgical timing, steroid initiation, infection status, blood markers, first tumor tissue	Diagnostic host-tumor state rather than long-latency causal exposure	Symptoms changing diet, sleep, medication use, inflammation, and healthcare contact	Subtype refinement and baseline immune/metabolic phenotyping
Treatment-period exposure	Radiotherapy dose and field, temozolomide, corticosteroids, antibiotics, antiepileptics, bevacizumab, serial MRI, blood or CSF samples	Therapy-induced immune, vascular, metabolic, and liquid-biopsy remodeling	Treatment intensity, performance status, infection, edema, steroid dose, assay timing	Response assessment, pseudoprogression interpretation, toxicity prevention, steroid tapering support
Recurrence-associated trajectory	Longitudinal MRI, liquid biopsy, metabolites, inflammatory proteins, treatment changes, reoperation tissue	Evolution of tumor and host biology under treatment pressure	Treatment effect, necrosis, inflammation, tumor shedding, variable sampling intervals	Recurrence monitoring and differentiation of progression from treatment effect
Survivorship and late effects	Neurocognitive outcomes, infections, metabolic status, sleep/activity, microbiome, medication burden, delayed imaging changes	Preventable morbidity and long-term host vulnerability	Survivor bias, changing supportive care, comorbidity, rehabilitation intensity	Survivorship protection, supportive-care personalization, late-toxicity monitoring

The table separates prediagnostic, peridiagnostic, treatment-period, recurrence-associated, and survivorship exposure windows to clarify whether a variable is being used for etiologic inference, diagnostic interpretation, response assessment, recurrence monitoring, toxicity prevention, or survivorship care.

This temporal distinction also explains why precision prevention in glioma should not be restricted to primary prevention. In common chronic diseases, exposure science often aims to prevent disease onset. In diffuse glioma, onset is rare, latency is long, and the strongest exposure evidence is limited. However, after diagnosis, the clinical pathway contains many preventable harms: infection, steroid toxicity, thromboembolism, metabolic deterioration, cognitive decline, delayed recurrence recognition, and unnecessary treatment for pseudoprogression. These harms occur in a measurable time window and can be linked to internal biomarkers, imaging, and clinical decisions. The postdiagnostic exposome is therefore not a secondary issue; it is one of the most practical entry points for exposure-aware precision prevention.

## 4 Exposure-sensitive pathways that reshape glioma ecology

### 4.1 Genotoxic and epigenetic remodeling

Genotoxic and epigenetic remodeling is the most direct conduit. Radiation illustrates this most clearly, but chemical mixtures, combustion products, oxidative stress, and treatment exposures may also influence DNA damage, repair, chromatin state, and methylation. Glioma is already a methylation- and chromatin-sensitive disease, as shown by IDH-driven hypermethylation, histone-defined subgroups, methylation signatures, and DNA repair-related treatment response (6, 12–14, 99). An exposure-linked epigenetic biomarker would not need to prove that an exposure initiated the tumor. It could still be useful if it identifies a biologically coherent subgroup, a treatment-response pattern, or a high-risk surveillance phenotype.

Single-cell work in IDH-mutant gliomas also shows why exposure biology cannot be interpreted without lineage and genotype. Genetics, lineage, and microenvironment can be decoupled, meaning that the same molecular driver can appear in different cellular and ecological contexts (100). This is important for exposome research because an exposure may interact with cell lineage, developmental state, immune niche, or treatment context rather than producing a uniform molecular signature. A pollution-linked inflammatory state, for example, might be relevant in vascular and macrophage-rich IDH-wildtype glioblastoma but not in the same way in oligodendroglioma. Similarly, radiation-associated epigenetic remodeling may differ by age at exposure and tumor developmental context.

### 4.2 Vascular injury, barrier dysfunction, and hypoxic niches

Vascular and barrier biology provides a second interface. External exposures such as pollution, radiation, systemic inflammation, metabolic disease, and treatment toxicity can plausibly affect endothelial state, blood-brain barrier permeability, edema, hypoxia, and perfusion. Human brain vascular multi-omics has begun to connect vascular biology to disease-risk associations, offering a broader methodological template for glioma research (101). In glioblastoma, spatial analysis and perfusion-linked studies show that vascular and hypoxic organization is not a background feature but a central component of tumor ecology (18, 51, 52). This makes vascular-barrier biology a plausible bridge between external exposure and spatial tumor state.

### 4.3 Neuroimmune regulation in myeloid-rich glioma ecosystems

Neuroimmune regulation provides a third interface and is central to the immunological relevance of an exposure-aware glioma framework. Glioma immune biology is shaped by microglia, monocyte-derived macrophages, myeloid-derived suppressor cells, T-cell exhaustion, tertiary lymphoid structures, hypoxic niches, antigen presentation, circadian immune regulation, and treatment-induced systemic immune suppression (50–52, 102–111). Exposures may influence this system through chronic inflammation, vascular injury, glucocorticoid signaling, microbiome-derived metabolites, infection, antibiotics, and metabolic stress. Corticosteroid exposure deserves particular attention because it can reduce edema and symptoms while also reshaping circulating lymphocytes, myeloid programs, cytokine measurements, imaging interpretation, and immunotherapy responsiveness. Exposure-aware immune biomarkers should therefore record steroid dose and timing, infection, antibiotic exposure, blood counts, inflammatory proteins, MRI features, and tissue immune states rather than interpreting tumor immune signatures in isolation.

### 4.4 Immunometabolic and microbiome-linked states

Immunometabolism links exposure, immune state, and glioma fitness. Microglial metabolic rewiring can shape immune-checkpoint blockade response, lipid-laden macrophages can recycle myelin to support glioblastoma, and metabolic competition can reinforce immune suppression (53, 54). Microbiome and intratumoral bacterial studies, together with Mendelian randomization analyses of gut microbiota, blood metabolites, and glioblastoma, suggest possible immune-metabolic interfaces (112–114). These findings remain preliminary in diffuse glioma, but they provide a tractable way to connect stool metagenomics, microbial metabolites, plasma metabolomics, cytokines, macrophage programs, and treatment tolerance. The most defensible near-term use is not microbiome-based glioma prevention, but immune-metabolic stratification in longitudinal cohorts and correlative immunotherapy studies.

### 4.5 Treatment-induced internal exposure after diagnosis

Treatment-induced internal exposure becomes dominant after diagnosis and is one of the most actionable parts of the framework. Radiotherapy, temozolomide, corticosteroids, antibiotics, antiepileptic drugs, bevacizumab, surgical injury, infection, rehabilitation, and recurrence-directed therapy can all alter immune tone, vascular permeability, edema, metabolism, microbiome composition, liquid-biopsy signal, and neurocognitive trajectory. These exposures are measurable perturbations rather than incidental background variables. They may explain why immune biomarkers, radiomics, metabolomics, and liquid-biopsy readouts differ across patients with similar tumor genotype. Corticosteroid- and antibiotic-associated immune remodeling is especially important because these exposures can confound immunotherapy biomarkers and may influence trial stratification.

**Figure 2:**
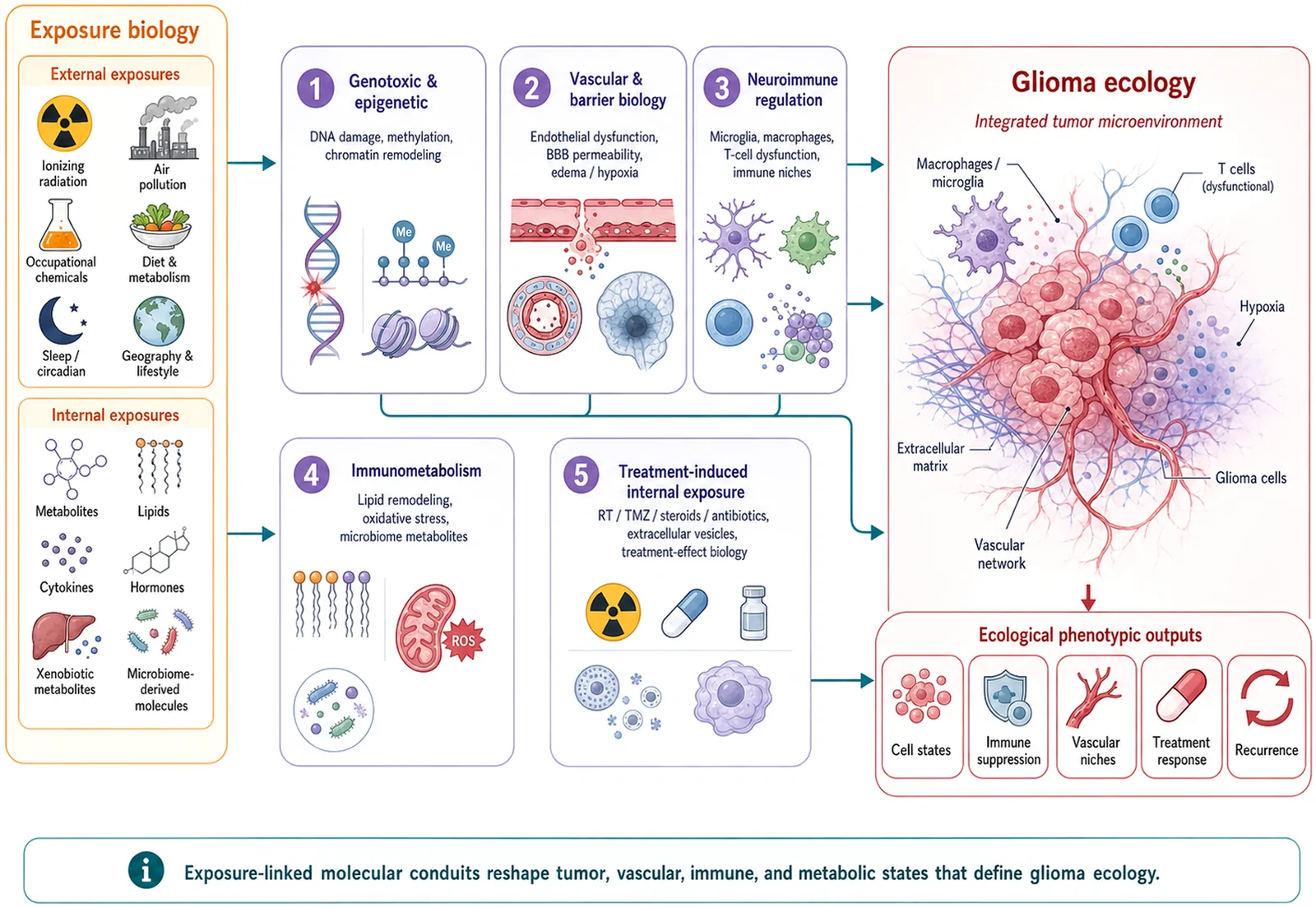
Biological conduits linking exposure biology to glioma ecology. Five interacting conduits are shown: genotoxic and epigenetic remodeling, vascular and blood-brain barrier dysfunction, neuroimmune regulation, immunometabolic and microbiome signaling, and treatment-induced internal exposure. Each conduit can be mapped to measurable tumor, host, imaging, or liquid-biopsy biomarkers.

**Figure 2** integrates these conduits as interacting routes through which exposure biology can reshape glioma ecology rather than as independent mechanisms.

## 5 Interpretable models for exposure-aware biomarker discovery

### 5.1 Time-anchored data architecture

Computational glioma exposomics begins with time-anchored data architecture rather than model complexity. External exposures may include residential geocoding, pollution models, job-exposure matrices, radiation records, diet, sleep, medication history, and treatment timelines. Internal exposures may include metabolomics, lipidomics, inflammatory proteins, microbiome features, xenobiotic markers, liquid biopsy, and MRI-derived phenotypes. Liquid-biopsy strategies for distinguishing progression, pseudoprogression, radiation necrosis, and extracellular-vesicle signals illustrate why biospecimen timing and clinical context should be encoded before modeling (115, 116). Each feature needs a prediagnostic, peridiagnostic, treatment, recurrence, or survivorship window before analysis. Practical barriers include small glioma cohorts, left-truncated exposure histories, nonrandom missingness, batch effects, site-specific MRI protocols, variable biospecimen timing, and confounding by treatment intensity and performance status.

### 5.2 Latent-factor discovery before supervised prediction

Interpretable discovery should precede highly flexible prediction. Exposome-wide association can screen external and internal exposures against subtype, immune state, recurrence, toxicity, or survival. Latent-factor approaches such as MOFA and MOFA+ can learn shared axes across omics layers, while LUCID can connect latent multi-omics clusters to phenotypic traits (117–119). Multi-omics profiling for health demonstrates how such integrative logic can be applied beyond a single disease platform (120). In glioma, interpretable latent factors might correspond to radiation-associated epigenetic remodeling, vascular-inflammatory hypoxia, macrophage-rich lipid metabolism, endocrine-metabolic state, or microbiome-linked immune suppression.

Supervised learning becomes useful after the biological objective and clinical decision are explicit. Graph neural networks can represent pathways, exposure mixtures, cell-cell communication, and spatial neighborhoods; multimodal deep learning can align imaging, pathology, liquid biopsy, and omics; and federated or site-aware learning may support multicenter development when data sharing is restricted. In practice, these models will often be limited by cohort size, missingness, exposure misclassification, and institutional variability. The most useful outputs are therefore interpretable clinical quantities, such as calibrated recurrence risk, probability of treatment effect versus progression, steroid-sensitive inflammatory state, toxicity-risk state, or trial-enrichment category, rather than opaque high-dimensional scores without a defined decision.

A practical AI workflow includes harmonized data dictionaries, explicit missingness maps, pre-specified time windows, separation of training and external validation cohorts, calibration assessment, decision-curve analysis, subgroup performance, and sensitivity analyses for exposure measurement error (121). Explainability should be biological and clinical, not only mathematical. A feature-importance plot is insufficient unless the model output can be linked to an exposure window, immune or metabolic pathway, tissue niche, liquid-biopsy marker, and management decision.

### 5.3 Causal and spatial validation

Causal and spatial validation are essential for biological credibility. Exposure science is vulnerable to confounding by geography, socioeconomic status, healthcare access, ancestry, occupation, treatment, and reverse causation. Causal diagrams, mediation analysis, negative controls, Mendelian randomization, target-trial emulation, and transportability assessment should be used whenever possible. The DEHP, benzo[a]pyrene, sleep, and microbiome-metabolite glioblastoma studies illustrate early causal-leaning strategies, but these findings require replication and biological validation (95–97, 114). Spatially resolved transcriptomic profiles of infiltrative 5ALA-metabolizing cells and single-cell/spatial transcriptomic studies of metabolic adaptations provide validation templates for localizing exposure-linked signatures in tissue (122, 123).

### 5.4 Clinical utility beyond model performance

The final tier is implementation science. A biomarker intended for discovery should be judged by reproducibility and pathway coherence. A biomarker intended for clinical use should be judged by calibration, decision-curve analysis, external validation, fairness, assay feasibility, and whether it changes management. Plasma-to-tumor integrated proteomics and blood-based assays for IDH1.R132H-mutant gliomas show how accessible biomarkers can be connected to tumor biology (71, 124). Successful validation of an exposure-aware biomarker should demonstrate temporal validity, assay reproducibility, causal interpretability where causal claims are made, and incremental value beyond age, performance status, molecular classification, extent of resection, MGMT promoter methylation, treatment regimen, and standard imaging.

A practical integration pipeline should also recognize that different layers serve different biological and clinical purposes. Germline information may capture inherited susceptibility and ancestry-related confounding. Tumor DNA and methylation capture relatively stable diagnostic biology. RNA, protein, and metabolite layers capture dynamic state. Single-cell and spatial data localize mechanisms to cell types and niches but are usually available for fewer patients. MRI provides longitudinal whole-lesion phenotypes but is affected by acquisition, segmentation, and treatment effect. Liquid biopsy provides repeated sampling but is influenced by tumor shedding, blood-brain barrier state, inflammation, and assay sensitivity. External exposure data often have broad coverage but high error. Integrative modeling should not flatten these layers into a single matrix without respecting their biological scale and timing. **Table 3** aligns major integration strategies with suitable inputs, validation requirements, and clinical decisions.

**Table 3:** Integration strategies for exposure-aware glioma biomarker development.

Integration task	Core inputs	Suitable methods	Validation priority	Clinical decision supported
Time-anchored data architecture	Exposure windows, treatment dates, MRI, biospecimens, tumor tissue	Common data model, harmonized dictionaries, missingness profiling	Temporal validity and transparent preprocessing	Define whether a variable is etiologic, diagnostic, therapeutic, or survivorship-related
Interpretable discovery	External exposure proxies, internal exposome, tumor omics, imaging	Exposome-wide association, MOFA/MOFA+, LUCID, sparse factor models	Pathway coherence, batch robustness, external replication	Identify host-tumor states for biomarker qualification
Supervised multimodal prediction	MRI, pathology, molecular markers, liquid biopsy, EHR, exposure time series	Regularized survival models, gradient boosting, multimodal deep learning	Calibration, discrimination, decision curves, external validation	Recurrence monitoring, response prediction, toxicity-risk estimation
Causal inference	Exposure data, confounders, outcomes, longitudinal clinical records	Directed acyclic graphs, mediation, negative controls, Mendelian randomization, target-trial emulation	Assumption checking, sensitivity analysis, transportability	Separate causal claims from prediction and prioritize modifiable pathways
Spatial and single-cell localization	Spatial transcriptomics, single-cell RNA-seq, multiplex imaging, histology	Cell-state mapping, niche analysis, ligand-receptor modeling, spatial statistics	Cell-type specificity and tissue localization	Validate whether exposure-linked signatures occur in relevant tumor niches
Liquid-biopsy qualification	Plasma, serum, CSF, extracellular vesicles, circulating tumor DNA, metabolites	Longitudinal mixed models, signal deconvolution, multi-analyte classifiers	Assay sensitivity, preanalytic control, relation to MRI and tissue	Distinguish recurrence, treatment effect, inflammation, and toxicity
Implementation and fairness	Multicenter cohorts, demographic variables, site factors, clinical outcomes	External validation, federated learning, subgroup calibration, decision analysis	Transportability, fairness, feasibility, actionability	Deploy biomarkers only where they improve a defined clinical action

The table aligns computational tasks with data inputs, suitable methods, validation priorities, and the clinical decisions they are intended to support.

A staged modeling strategy follows from these constraints. First, define the clinical endpoint and time window. Second, select exposure variables that are meaningful for that window. Third, use interpretable latent factors to identify candidate host-tumor states. Fourth, test whether supervised models improve clinically relevant prediction beyond standard variables. Fifth, perform causal sensitivity analysis and external validation. Sixth, localize the signal spatially or experimentally. This staged approach is more conservative than immediate large-scale neural-network modeling, but it is more likely to yield a temporally valid, biologically interpretable, and clinically testable biomarker.

## 6 Biomarker opportunities along the glioma disease trajectory

### 6.1 High-risk surveillance rather than population screening

High-risk surveillance is the most plausible prediagnostic application, not population screening. Prior cranial irradiation, cancer survivorship, familial predisposition, and intense occupational exposure are more plausible settings for surveillance research than the general population. In such cohorts, exposure-aware biomarkers could combine radiation history, geospatial exposure, occupational history, plasma proteins, metabolites, methylation signatures, MRI changes, and eventual tumor molecular data. The endpoint would be risk stratification and early evaluation of concerning imaging changes, not a universal blood test for glioma.

### 6.2 Molecular subtype refinement through host-tumor states

Subtype refinement is a second use case. WHO diagnosis, IDH status, 1p/19q codeletion, TERT promoter mutation, histone alterations, MGMT promoter methylation, and methylation class remain foundational (3, 6, 11, 12). Exposome-multi-omics should not replace them. Instead, it can layer host-tumor states on top of them: radiation-associated epigenetic state, pollution-linked vascular inflammation, plasticizer-linked S100A4/lipid biology, microbiome-metabolite immune suppression, allergy-associated immune tone, or steroid-antibiotic metabolic dysregulation. Such states would need external validation before they could be used clinically.

### 6.3 Response assessment and recurrence monitoring

Treatment response and recurrence monitoring form a third use case. Proteometabolomics, metabolic remodeling, radiomics, and multimodal integration indicate that recurrence is not simply the reappearance of the baseline tumor (41, 44, 59, 60). Treatment alters the tumor and host environment. Liquid biopsy can help track tumor evolution, extracellular vesicles, and circulating biomarkers (64–70). Adding internal exposome markers may help separate tumor evolution from inflammation, treatment effect, metabolic stress, or medication exposure.

Response interpretation is a related clinical problem. Neuro-oncology frequently faces ambiguous radiographic change after radiotherapy and temozolomide. Enhancement, edema, necrosis, immune activation, vascular injury, steroid response, and true tumor growth can overlap. A tumor-only model may interpret ambiguity as insufficient imaging resolution. An exposure-aware model can add the missing context of radiation dose and timing, steroid exposure, infection, inflammatory markers, liquid-biopsy kinetics, metabolic stress, and vascular features. This does not eliminate uncertainty, but it can make uncertainty more structured. The goal is not to replace expert neuroradiology; it is to provide a biologically informed context in which imaging and molecular data can be interpreted together.

### 6.4 Immunotherapy stratification and survivorship protection

Immunotherapy and trial stratification provide a fourth use case. Glioblastoma immunotherapy has been difficult, partly because the immune microenvironment is heterogeneous, suppressive, and spatially structured. Immune suppression in gliomas, immunopathology of glioblastoma, and glioma macrophage studies show that immune biomarkers must be cell-type-specific and context-aware (50, 104, 125, 126). Exposure-aware biomarkers could identify macrophage-rich hypoxia, lipid-laden myeloid ecosystems, tertiary lymphoid structures, steroid-associated systemic immune suppression, antibiotic- or microbiome-linked metabolic states, and cytokine-protein profiles that affect immune monitoring (127). These biomarkers would be most useful as trial-enrichment or correlative markers, not as stand-alone predictors of immunotherapy benefit.

Survivorship and toxicity prevention may be the most practical near-term use case. Precision prevention in glioma should include prevention of avoidable harm after diagnosis: steroid complications, infection, metabolic decline, neurocognitive deterioration, delayed recognition of recurrence, and unnecessary interventions for treatment effect. Exposure-aware models could combine treatment exposures, liquid biopsy, imaging, inflammatory markers, sleep, diet, microbiome, and activity to identify modifiable risk states. This is a more realistic near-term use of precision prevention than claiming that common environmental exposures can prevent sporadic glioma onset.

Clinical translation will depend on matching each biomarker to a realistic decision. A high-risk surveillance biomarker should be stable enough for repeated measurement and specific enough to avoid unnecessary imaging or anxiety. A recurrence-monitoring biomarker should change over time and add information beyond conventional MRI. A treatment-response biomarker should be available before the therapeutic decision. A toxicity-prevention biomarker should identify a modifiable host state before irreversible decline. These distinctions matter because many proposed glioma biomarkers are biologically interesting but clinically underspecified. Exposure-aware biomarkers may be especially useful when they clarify decisions that are already difficult in practice, including steroid tapering, follow-up interval selection, trial enrichment, and evaluation of post-treatment enhancement.

Assay selection should follow the intended clinical use. A high-risk surveillance biomarker should be inexpensive, repeatable, and robust to common inflammatory conditions. A recurrence-monitoring biomarker should be sensitive to tumor evolution but specific enough to avoid overcalling pseudoprogression. A trial-stratification biomarker can be more complex if it enriches for a mechanistically defined subgroup. A toxicity-prevention biomarker should be available before irreversible harm occurs. This means that some discovery layers, such as spatial transcriptomics, may be unsuitable for routine monitoring but excellent for validating a liquid-biopsy or imaging marker. A mature exposome-multi-omics field will therefore use different assays at discovery, qualification, and implementation stages rather than expecting one assay to answer every question.

### 6.5 Biomarker terminology by clinical use

Biomarker terminology should be precise. A risk marker identifies a probability state; a predictive marker identifies differential treatment benefit; a monitoring marker tracks change over time; a mechanistic marker points to a pathway; and a prevention marker supports an intervention that reduces harm. Many glioma signatures are called biomarkers without specifying which category they belong to. Biomarkers should therefore be labeled by use case. Such precision prevents overstated translational claims.

## 7 Precision prevention across the glioma care continuum

### 7.1 Primary and secondary prevention in defined risk settings

Primary prevention requires cautious framing. The most defensible actions are broad public-health and clinical-safety measures: avoid unnecessary ionizing radiation, optimize radiation protection, improve occupational chemical safety, reduce harmful air pollution, and support general metabolic and immune health. These actions have benefits beyond glioma and do not require overstating glioma-specific causal evidence. Current exposome evidence does not justify lifestyle-based or exposure-based screening of the general population for sporadic glioma. Precision prevention is used here mainly for high-risk surveillance, earlier recognition of adverse trajectories, reduction of treatment-related immune and metabolic harm, recurrence-aware monitoring, and survivorship protection.

Secondary prevention is most credible in defined high-risk groups. Survivors exposed to cranial irradiation, individuals with genetic predisposition, patients with unusual exposure histories, and selected occupational groups could be enrolled in prospective registries. These registries should collect exposure history, geospatial variables, biospecimens, MRI, and molecular data when tissue becomes available. Computational models could then test whether exposure-linked latent factors improve risk stratification beyond conventional variables.

### 7.2 Tertiary prevention during treatment and survivorship

Tertiary precision prevention is the strongest near-term application. After diagnosis, the clinical goal is to prevent avoidable deterioration and detect adverse trajectories early. A patient receiving chemoradiation is exposed to radiation, temozolomide, steroids, antibiotics, antiepileptic drugs, inflammation, sleep disruption, nutritional change, and psychological stress. These exposures may influence immune state, metabolism, infection risk, edema, neurocognition, and recurrence assessment. A model that integrates these exposures with MRI and liquid biopsy could support surveillance intervals, supportive-care interventions, and trial stratification.

### 7.3 Prospective pilots for decision support

**Figure 3:**
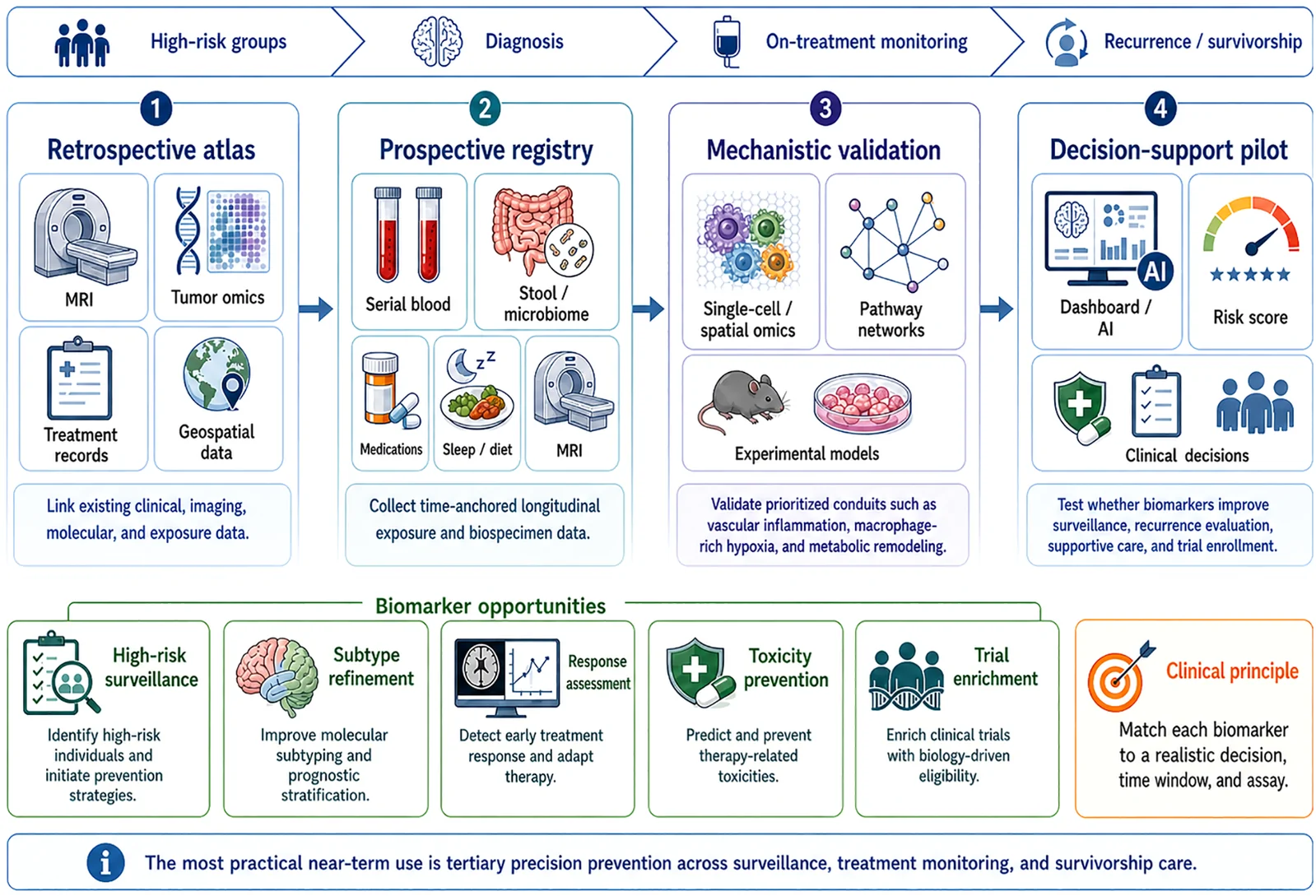
Translational roadmap for exposome-multi-omics biomarker discovery and precision prevention. The roadmap moves from retrospective atlas construction to prospective longitudinal registries, mechanistic validation, and decision-support pilots. Clinical applications include high-risk surveillance, response assessment, recurrence monitoring, toxicity prevention, and trial stratification.

A translational research program can proceed in four stages. Stage one is a retrospective atlas linking existing MRI, clinical records, tumor omics, treatment exposures, and geospatial data. Stage two is a prospective registry with serial blood, stool, MRI, medication, sleep, diet, and outcome data. Stage three is mechanistic validation of prioritized conduits such as radiation-epigenetic remodeling, pollution-vascular inflammation, DEHP-S100A4 lipid biology, microbiome-metabolite immune suppression, and macrophage-rich hypoxia. Stage four is a decision-support pilot that tests whether the biomarker changes surveillance, supportive care, recurrence evaluation, or trial enrollment. This staged program is ambitious but feasible, and it follows the central framework rather than drifting into an unfocused literature catalogue. **Figure 3** translates this program into a disease-trajectory roadmap.

A first pilot could focus on newly diagnosed IDH-wildtype glioblastoma because tissue, MRI, treatment, and recurrence data are usually available. The pilot would collect baseline tumor methylation and transcriptomics, pre- and post-chemoradiation plasma metabolomics and inflammatory proteins, medication exposures, steroid dose, antibiotic exposure, geocoded pollution estimates, and serial MRI. The primary analysis would test whether exposure-linked latent factors explain recurrence timing or treatment effect beyond age, performance status, extent of resection, MGMT methylation, and standard imaging. A second pilot could focus on post-radiation survivors because exposure timing and dose are better defined. These focused pilots are more likely to succeed than an all-exposures, all-omics study without a clear endpoint.

## 8 Discussion

Exposure measurement and causal interpretation remain the main constraints on exposure-aware glioma research. Residential address, job title, diet questionnaire, and self-reported device use are practical but noisy proxies, whereas metabolites, lipids, cytokines, microbiome products, and xenobiotic markers are more proximal but can be altered by tumor burden, therapy, medication, infection, and behavior. Prediagnostic exposure, diagnostic biology, perioperative stress, chemoradiation exposure, recurrence biology, and survivorship exposure should therefore be modeled as distinct windows rather than pooled as interchangeable covariates. Geography, socioeconomic status, healthcare access, ancestry, occupation, treatment pattern, and performance status can all confound exposure-outcome relationships, and prediagnostic symptoms may themselves change diet, sleep, medication use, and healthcare contact. Causal diagrams, negative controls, mediation analysis, Mendelian randomization, target-trial emulation, and sensitivity analysis can strengthen inference, but causal language should remain conservative unless exposure timing, confounding structure, biological validation, and reproducibility are aligned.

Reproducibility will depend on multicenter design and transparent reporting. Glioma is rare, and high-dimensional omics are expensive, making single-center black-box models with many exposure and omics features especially vulnerable to overfitting and poor transportability. Studies should report exposure domains and time windows, missingness, batch correction, training and validation separation, calibration, discrimination, decision-curve analysis, subgroup performance, and fairness metrics. For imaging, acquisition, segmentation, preprocessing, and external validation should be explicit; for multi-omics, platform details, normalization, batch correction, and feature selection should be documented; and for liquid biopsy, biospecimen timing, processing, assay sensitivity, and clinical context should be specified. These requirements are not technical ornamentation. They determine whether exposure-aware biomarkers can be reproduced across institutions and populations.

Clinical anchoring is equally important. A discovery signature may be biologically informative without being ready for practice, but a prevention biomarker must ultimately support a decision that clinicians already face, such as whether a high-risk survivor needs closer imaging, whether post-treatment enhancement is more consistent with recurrence or treatment effect, whether steroid exposure can be safely reduced, whether infection or metabolic deterioration is emerging, or whether a patient should enter a mechanistically enriched trial. Each use case has a different tolerance for false-positive and false-negative results, and each requires comparison with standard clinical, imaging, and molecular variables. Long catalogues of exposures are therefore less useful than exposure-linked states that connect measurable internal biology, immune or metabolic mechanism, cell type, tissue niche, longitudinal sampling, and a defined clinical action.

The central argument is not that artificial intelligence can predict glioma better in isolation, but that computational integration can connect exposure, host state, immune microenvironment, tumor ecology, and preventable clinical trajectories when used with mechanistic discipline. Two interpretive risks should be avoided. One is fragmentation, in which artificial intelligence, multi-omics, radiomics, immunology, epidemiology, and exposome science remain adjacent but disconnected literatures. The other is overclaiming, in which pollution, diet, pesticides, plasticizers, microbiome variation, or sleep disruption are described as established glioma causes despite incomplete evidence. Diffuse glioma is ready for exposure-aware systems neuro-oncology because the field already has molecular classification, radiomics, single-cell atlases, spatial biology, immunometabolism, and liquid biopsy. What remains underdeveloped is the integration of those layers with external and internal environments that shape host-tumor immune trajectories before and after diagnosis. The exposome should not be used to justify unsupported population-level prevention claims; it should be used to discover biologically interpretable biomarkers for high-risk surveillance, treatment response, recurrence monitoring, toxicity prevention, immunotherapy stratification, and rational trial design.
